# Potassium stimulates fruit sugar accumulation by increasing carbon flow in *Citrus sinensis*

**DOI:** 10.1093/hr/uhae240

**Published:** 2024-09-09

**Authors:** Kongjie Wu, Chengxiao Hu, Peiyu Liao, Yinlong Hu, Xuecheng Sun, Qiling Tan, Zhiyong Pan, Shoujun Xu, Zhihao Dong, Songwei Wu

**Affiliations:** Hubei Provincial Engineering Laboratory for New Fertilizers/Key Laboratory of Arable Land Conservation (Middle and Lower Reaches of Yangtze River), Ministry of Agriculture and Rural Affairs, Huazhong Agricultural University, Shizhishan Street, Hongshan District, Wuhan, Hubei, 430070 China; National Key Laboratory for Germplasm Innovation & Utilization of Horticultural Crops, Huazhong Agricultural University, Shizhishan Street, Hongshan District, Wuhan, Hubei, 430070 China; Hubei Provincial Engineering Laboratory for New Fertilizers/Key Laboratory of Arable Land Conservation (Middle and Lower Reaches of Yangtze River), Ministry of Agriculture and Rural Affairs, Huazhong Agricultural University, Shizhishan Street, Hongshan District, Wuhan, Hubei, 430070 China; National Key Laboratory for Germplasm Innovation & Utilization of Horticultural Crops, Huazhong Agricultural University, Shizhishan Street, Hongshan District, Wuhan, Hubei, 430070 China; Hubei Provincial Engineering Laboratory for New Fertilizers/Key Laboratory of Arable Land Conservation (Middle and Lower Reaches of Yangtze River), Ministry of Agriculture and Rural Affairs, Huazhong Agricultural University, Shizhishan Street, Hongshan District, Wuhan, Hubei, 430070 China; National Key Laboratory for Germplasm Innovation & Utilization of Horticultural Crops, Huazhong Agricultural University, Shizhishan Street, Hongshan District, Wuhan, Hubei, 430070 China; Hubei Provincial Engineering Laboratory for New Fertilizers/Key Laboratory of Arable Land Conservation (Middle and Lower Reaches of Yangtze River), Ministry of Agriculture and Rural Affairs, Huazhong Agricultural University, Shizhishan Street, Hongshan District, Wuhan, Hubei, 430070 China; National Key Laboratory for Germplasm Innovation & Utilization of Horticultural Crops, Huazhong Agricultural University, Shizhishan Street, Hongshan District, Wuhan, Hubei, 430070 China; Hubei Provincial Engineering Laboratory for New Fertilizers/Key Laboratory of Arable Land Conservation (Middle and Lower Reaches of Yangtze River), Ministry of Agriculture and Rural Affairs, Huazhong Agricultural University, Shizhishan Street, Hongshan District, Wuhan, Hubei, 430070 China; Hubei Provincial Engineering Laboratory for New Fertilizers/Key Laboratory of Arable Land Conservation (Middle and Lower Reaches of Yangtze River), Ministry of Agriculture and Rural Affairs, Huazhong Agricultural University, Shizhishan Street, Hongshan District, Wuhan, Hubei, 430070 China; National Key Laboratory for Germplasm Innovation & Utilization of Horticultural Crops, Huazhong Agricultural University, Shizhishan Street, Hongshan District, Wuhan, Hubei, 430070 China; Guangdong Agricultural Environment and Cultivated land Quality Protection Center, Huanshizhong Street, Yuexiu District, Guangzhou 510599 China; Hubei Provincial Engineering Laboratory for New Fertilizers/Key Laboratory of Arable Land Conservation (Middle and Lower Reaches of Yangtze River), Ministry of Agriculture and Rural Affairs, Huazhong Agricultural University, Shizhishan Street, Hongshan District, Wuhan, Hubei, 430070 China; Hubei Provincial Engineering Laboratory for New Fertilizers/Key Laboratory of Arable Land Conservation (Middle and Lower Reaches of Yangtze River), Ministry of Agriculture and Rural Affairs, Huazhong Agricultural University, Shizhishan Street, Hongshan District, Wuhan, Hubei, 430070 China; National Key Laboratory for Germplasm Innovation & Utilization of Horticultural Crops, Huazhong Agricultural University, Shizhishan Street, Hongshan District, Wuhan, Hubei, 430070 China

## Abstract

Soluble sugars contribute to the taste and flavor of citrus fruit. Potassium (K), known as a quality element, plays key roles in improving sugar accumulation and fruit quality, but the mechanism is largely unknown. This study aims to elucidate how K improves sugar accumulation by regulating carbon flow from source leaves to fruit in Newhall navel orange. We found that optimal fruit K concentrations around 1.5% improved sugar accumulation and fruit quality in citrus. K application increased the strength of both sink and source, as indicated by the increased fruit growth rate, enzyme activities and expression levels of key genes involved in sucrose (Suc) metabolism in fruit and leaf. K application also facilitated Suc transport from source leaves to fruit, as confirmed by the enhanced ^13^C-Suc level in fruit. Furthermore, we found that navel orange used the symplastic pathway for transporting Suc from source leaves to fruit, and K application enhanced symplastic loading, as demonstrated by the intensified carboxyfluorescein signal and increased plasmodesmata density in leaves. The findings reveal that K stimulates fruit sugar accumulation by increasing carbon flow from source leaves to fruit in Newhall navel orange.

## Introduction

Potassium (K) is an essential mineral element for plants, playing a crucial role in plant growth and development [1]. K predominantly exists in the form of K^+^, exhibiting multiple physiological functions, including regulation of photosynthesis, improvement of carbohydrate transport, increment of source carbohydrate synthesis, and maintenance of cytoplasmic pH homeostasis [2–4]. K influences cell swelling pressure, which is positively correlated with fruit sugar content and hardness [5, 6]. K promoted photosynthesis in source leaves, thus enhancing sugar metabolism and accumulation in sink organs [7], and the reduction in K supply can decrease carbohydrate allocation to roots [8]. The sugar content in fruit is primarily determined by the transport of carbohydrates from source leaves to fruits and sucrose (Suc) metabolism within the fruit itself [9, 10]. K increases sugar accumulation in fruit through regulation of Suc metabolism, but there are still limited reports on how K promotes carbohydrate transport in fruit tree plants and enhances sugar content. The mechanisms underlying K-promoted carbohydrate transport have been documented in annual plants; K improves carbohydrate transport mainly by increasing osmotic pressure [11–13] and ATP energy [14, 15]. However, there are few reports on the promotion of carbohydrate transport by K in citrus, a perennial woody plant. Investigating the mechanism by which K mediates carbohydrate transport is crucial for enhancing citrus fruit quality.

Source strength refers to the capacity of a plant to synthesize and allocate carbohydrate, which is directly determined by the efficiency of photosynthesis [16, 17]. Sink strength refers to the capacity of sink organs to compete for photosynthetic products, and it is calculated as the product of sink size and sink activity. Sink activity denotes the carbohydrate uptake rate, which is determined by Suc transport in phloem and its metabolic capacity in sink organs [18, 19]. Photosynthetic leaves serve as the primary source organs in plants, and their morphological characteristics and photosynthetic intensity largely influence the source strength [20]. Shading has been reported to lead to a significant reduction in total soluble sugar content in the leaves of navel oranges [21]. Sink strength determines the pressure gradient from source leaves to sink, thereby regulating long-distance transport of carbohydrates from source leaves to fruit [22, 23]. The transport of photosynthetic products from source leaves to sink organs in the form of Suc requires both symplastic loading and apoplastic loading, and this transport is conducive to plant source–sink balance maintenance [24]. Suc is primarily transported from mesophyll cells of source leaves to parenchymal cells of phloem via plasmodesmata, which is known as the symplastic pathway [25, 26]. However, some plants, such as melon [27], maize [28], and potato [29], export Suc from parenchymal cells to the apoplastic space through SWEET protein, and subsequently Suc is absorbed into the sieve molecule-associated cell complex by Suc transporters (SUTs) and transferred to sink organs, which is known as the apoplastic pathways [24, 30]. In addition to symplastic loading and apoplastic loading working alone, these two phloem loading systems can synergistically act in response to carbohydrate transport and allocation between source and sink organs [31–35]. However, few studies have been conducted to explore whether K affects sugar accumulation in fruit by regulating symplastic and apoplastic phloem loading in citrus.

The market competitiveness of citrus is determined by fruit quality, and enhancing fruit quality has become a prominent concern in the field of pomology [36]. The deficiency and excess of K in the soil of citrus orchards can easily lead to the deterioration of fruit flavor quality, thereby constraining the sustainable development of the citrus industry [37, 38]. Our previous study has demonstrated that K application effectively enhances the sugar content in citrus fruits [4]. However, the underlying mechanism by which K regulates carbohydrate transport to promote sugar accumulation in citrus fruit remains elusive. In this study, we hypothesized that K might influence source and sink strength by regulating carbohydrate transport in the citrus plant. To test this hypothesis, we investigated the effects of different amounts of K fertilizer on source strength, sink strength, and Suc phloem transport in 10-year-old Newhall navel oranges (*Citrus sinensis*) through field and pot experiments. The results showed that K stimulated carbon flow between source and sink via the modulation of symplastic loading pathways, thereby enhancing soluble sugar accumulation in citrus fruit. Our revealed mechanism underlying K-mediated sugar accumulation in fruit provides the theoretical basis for enhancing citrus fruit quality.

## Results

### Potassium application improves fruit yield and quality of Newhall navel orange

**Figure 1:**
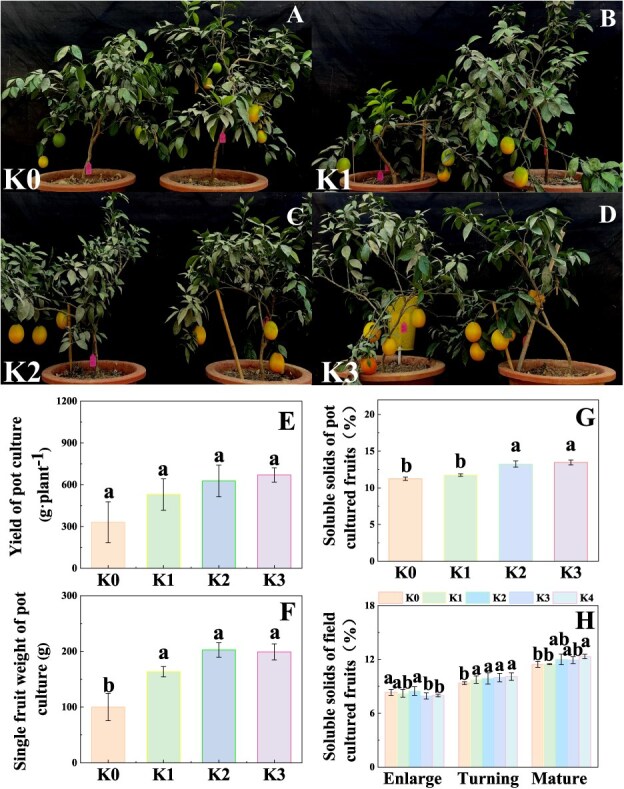
Effects of K fertilizer on fruit yield and quality in Newhall navel orange. **A**–**D** Pot culture. **E** Yield of pot culture. **F** Single fruit weight of pot culture. **G** Soluble solids of pot-cultured fruits. K0, K1, K2, and K3 indicate that the citrus plants were cultivated with K fertilizer levels of 0, 0.20, 0.40, and 0.60 g K_2_O kg^−1^ soil under pot culture. **H** Field culture, soluble solids of field cultured fruits. K0, K1, K2, K3, and K4 indicate that the citrus plants were cultivated in different K fertilizer levels of 0, 0.25, 0.5, 0.75, 0.9 kg K_2_O per plant under field culture. Data and error bars are mean ± standard error (*n* = 4), and different lowercase letters represent significant differences among K treatments at the same stage by Duncan’s test (*P <* 0.05).

Pot experiment results showed that K application induced fruit enlargement and color turning, which was consistent with the field experiment results that K application increased the single fruit weight, fruit number per plant, and yield of Newhall navel orange (Fig. 1A–F, Supplementary Data Table S2). Compared with K0 treatment (no K applied), K application significantly increased the contents of total soluble solid (TSS) in Newhall navel orange at the mature stage under pot and field experiments (Fig. 1G and H). The K content in leaves was significantly higher in K application groups than that in K0 treatment group at the enlargement and mature stages in field experiment. Similarly, the K content in fruit pulp was increased by K application at the mature stage (Supplementary Data Fig. S1C). These results showed that an appropriate amount of K improved fruit yield and quality in Newhall navel orange.

### Potassium application improves soluble sugar accumulation in Newhall fruit

**Figure 2:**
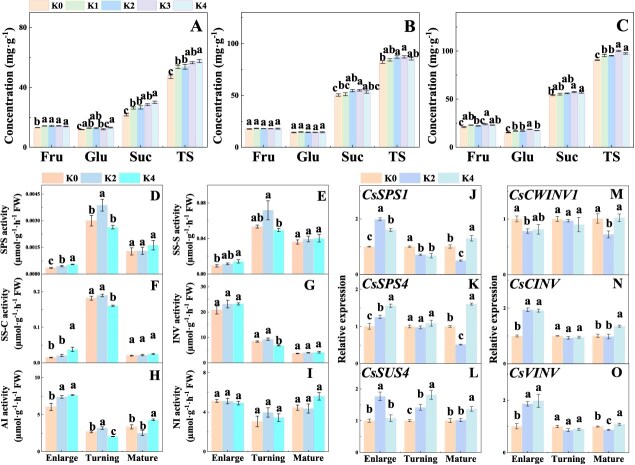
Effects of K fertilizer on soluble sugar concentrations and Suc metabolism in Newhall navel orange fruit. **A** Enlargement stage. **B** Color turning stage. **C** Mature stage. Fru, Glu, Suc, and TS represent sucrose, fructose, glucose, sucrose, and total sugars, respectively. **D** Sucrose phosphate synthase (SPS). **E** Sucrose synthetase synthesis (SS-S). **F** Sucrose synthetase cleavage (SS-C). **G** Invertase (INV). **H** Acid invertase (AI). **I** Neutral invertase (NI). **J**–**O** Expression of Suc-metabolizing enzyme genes. K0, K1, K2, K3, and K4 indicate that the citrus plants were cultivated with K fertilizer levels of 0, 0.25, 0.5, 0.75, 0.9 kg K_2_O per plant under field culture. Data and error bars are mean ± standard error (*n* = 4), and different lowercase letters represent significant differences among K treatments at the same stage by Duncan’s test (*P <* 0.05).

Suc, fructose (Fru), and glucose (Glu) are the main components of soluble sugar in citrus fruit [4], and the concentrations of Suc, Fru, Glu, and total sugars were significantly increased by K application during the development stage of Newhall navel orange (Fig. 2A–C). Specifically, at the stage of enlargement, the Suc concentration was 20.38, 22.31, 31.08, and 38.52% higher under K1, K2, K3, and K4 treatments than that under K0 treatment, respectively; at the stage of color turning, K2 and K3 treatments resulted in an increase of 8.40 and 9.19% in Suc concentration, respectively; at the mature stage, K3 and K4 treatments led to an increase of 6.11 and 5.02% in Newhall navel orange. As for total sugar concentration, at the stage of enlargement, it was significantly increased by 13.79, 14.25, 19.95 and 22.34% under K1, K2, K3, and K4 treatments, respectively; at the stage of color turning, by 5.90 and 3.80% under K3, and K4 treatments; and at the mature stage, by 5.31, 5.14, 10.75, and 7.70% under K1, K2, K3, and K4 treatments. To further elucidate the relationship between sugar concentrations and K concentrations in fruit, we performed regression analysis. The results showed that Suc concentration was significantly correlated with K concentration in fruit pulp (Supplementary Data Fig. S3). Moreover, 1.50% K concentration in pulp was the critical value beyond which Suc concentration in fruit decreased due to excessive K application.

### Potassium application improves sucrose metabolism and sink strength in Newhall fruit

Compared with K0 treatment, K2 and K3 treatments dramatically increased fruit size and the average growth rate of fruit, and K4 treatment dramatically increased fruit number (Supplementary Data Table S2). Compared with K0 treatment, K2 and K4 treatments increased the activities of the Suc-synthesizing enzyme sucrose phosphate synthase (SPS) and sucrose synthetase synthesis (SS-S). Specifically, at the stage of enlargement, K2 and K4 treatments significantly increased SPS activity but only K4 treatment significantly increased SS-S activity, and at the stage of color turning, K2 treatment significantly increased SPS activity (Fig. 2D and E). In addition, at the stage of enlargement, sucrose synthetase cleavage (SS-C) activity was significantly increased under K4 treatment and acid invertase (AI) activity under both K2 and K4 treatments (Fig. 2F and H), but at the stage of color turning, the activities of SS-C and invertase (INV) were dramatically inhibited under K4 treatment (Fig. 2F and G).

At the enlargement stage, compared with expressions under K0 treatment, the expressions of *CsSPS1* and *CsSPS4* genes were significantly increased under K2 and K4 treatments, whereas at the mature stage, the expressions of these two genes were markedly decreased under K2 treatment (Fig. 2J and K). Similarly, at the enlargement and color turning stages, the expression of *CsSUS4* was significantly increased under K2 and/or K4 treatments (Fig. 2L). Furthermore, at the enlargement stage, the expressions of *CsCINV* and *CsVINV* were markedly increased under K2 and K4 treatments, but at the color turning stage, no significant differences in the expression levels of *CsCWINV1*, *CsCINV*, and *CsVINV* were observed among the treatments (Fig. 2M–O).

### Potassium application improves the transport of sugar in Newhall fruit

**Figure 3:**
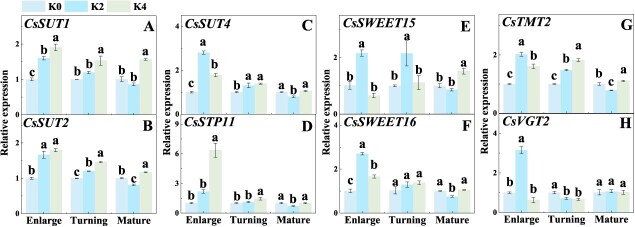
Effects of K fertilizer on the expressions of sugar transporters in Newhall navel orange fruit. K0, K2, and K4 indicate that the citrus plants were cultivated with K fertilizer levels of 0, 0.5, and 0.9 kg K_2_O per plant under field culture. Data and error bars are mean ± standard error (*n* = 4), and different lowercase letters represent significant differences among K treatments at the same stage by Duncan’s test (*P <* 0.05).

At the enlargement stage, the expressions of *CsSUT1*, *CsSUT2*, *CsSUT4*, *CsSWEET16*, and *CsTMT2* were significantly increased under both K2 and K4 treatments compared with K0 treatment, but the expressions of *CsSWEET15* and *CsVGT2* were significantly induced only under K2 treatment (Fig. 3). At the color turning stage, *CsSUT2*, *CsSUT4*, and *CsTMT2* expression levels were significantly upregulated under both K2 and K4 treatments, while *CsSUT1* and *CsSTP11* expression levels were significantly upregulated only under K4 treatment, and *CsSWEET15* was significantly upregulated only under K2 treatment in Newhall navel orange fruit (Fig. 3).

### Potassium application improves sugar metabolism and source strength in Newhall leaf

**Figure 4:**
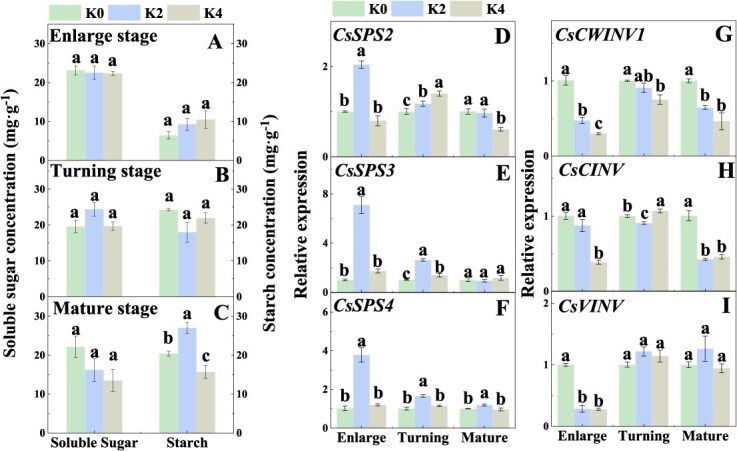
Effects of K fertilizer on the concentrations of soluble sugar and starch and the expression levels of Suc-metabolizing enzyme genes in Newhall orange leaf. K0, K1, K2, K3, and K4 indicate that the plants were cultivated with K fertilizer levels of 0, 0.25, 0.5, 0.75, and 0.9 kg K_2_O per plant under field culture. Data and error bars are mean ± standard error (*n* = 4), and different lowercase letters represent significant differences among K treatments at the same stage by Duncan’s test (*P <* 0.05).

Since source strength determines the amount of carbohydrate exported [39], we measured the effects of K application on leaf sugar metabolism to further comprehend the reason for the increase in soluble sugar in fruit. The results showed that the transpiration rate (*T*_r_) and stomatal conductance (*G*_s_) of orange leaves were significantly higher under K1 and K2 treatments than those under K0 treatment. Similarly, the net photosynthetic rate (*P*_n_) of orange leaves was significantly increased by 43.08–84.57% under different amounts of K treatment (Supplementary Data Table S3), indicating that K application improved the fixation of carbon. Moreover, at the enlargement stage, Suc-synthesizing enzyme SPS activity was significantly increased under K2 treatment, while at the color turning stage, SS-S activity was significantly increased under both K2 and K4 treatments (Supplementary Data Fig. S4). At the stages of color turning and mature, the activity of AI was significantly inhibited under K2 and K4 treatments (Supplementary Data Fig. S4). Compared with those under K0 treatment, the expressions of *CsSPS2*, *CsSPS3*, and *CsSPS4* genes were significantly upregulated under K2 treatment at enlargement and color turning stages, but the expressions of *CsCWINV1*, *CsCINV*, and *CsVINV* were significantly downregulated at enlargement and mature stages (Fig. 4D–I). At enlargement and color turning stages, the expressions of *CsSTP7* and *CsTMT1* were significantly higher under K2 and K4 treatments than those under K0 treatment (Supplementary Data Fig. S5). The above results indicated that K application improved Suc synthesis in Newhall orange leaves at the enlargement and mature stages. However, no significant differences in soluble sugar and starch concentrations were observed among K treatments during the period of fruit development (Fig. 4A–C), suggesting that K application may promote Suc transport from source leaves to fruits.

### Potassium application improves carbohydrate transport from source to sink in Newhall orange

**Figure 5:**
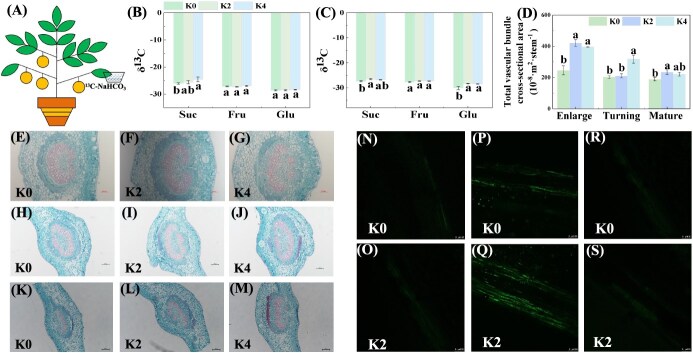
Effects of K fertilizer on carbon flow by phloem transport in Newhall orange leaf to fruit. K0, K2, and K4 indicate that the citrus plants were cultivated with K fertilizer levels of 0, 0.5, and 0.9 kg K_2_O per plant under field culture. **A**  ^13^C-NaHCO_3_ labeling experimental model. **B**, **C**  ^13^C-Sugar components in fruit: enlargement stage (**B**); color turning stage (**C**). **D** Total cross-sectional area of vascular bundle of leaf vein. **E**–**M** Cross-sections of vascular bundles of leaf veins: enlargement stage (**E**–**G**); color turning stage (**H**–**J**); mature stage (**K**–**M**). **N**–**S** CF fluorescence signal in phloem: enlargement stage (**N**, **O**); color turning stage (**P**, **Q**); mature stage (**R**, **S**). Data and error bars are mean ± standard error (*n* = 4), and different lowercase letters represent significant differences among K treatments at the same stage by Duncan’s test (*P <* 0.05).

Carbohydrate transport plays crucial roles in the accumulation of soluble sugars in fruit [9] and half of the sugars in citrus fruit are derived from carbohydrate transport [40]. To examine the roles of K in regulating carbohydrate transport in citrus, we performed a ^13^C-isotope experiment (Fig. 5A). The results showed that K application significantly increased the concentrations of ^13^C-Suc in orange fruit at the enlargement stage, and the concentrations of ^13^C-Suc and ^13^C-Glu at the color turning stage, suggesting that K application promoted sugar transport from source leaves to sink fruits (Fig. 5B and C).

**Figure 6:**
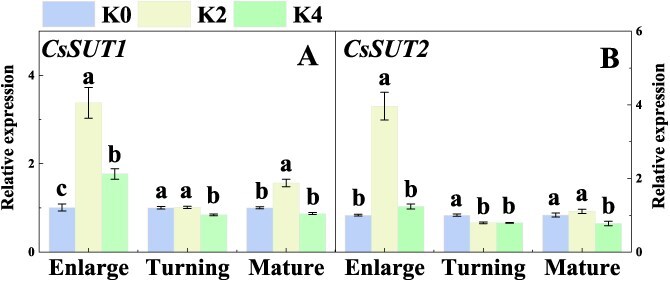
Effects of K fertilizer on the expression of sugar transporter in Newhall orange leaf. K0, K2, and K4 indicate that the citrus plants were cultivated with K fertilizer levels of 0, 0.5, and 0.9 kg K_2_O per plant under field culture. Data and error bars are mean ± standard error (*n* = 4), and different lowercase letters represent significant differences among K treatments at the same stage by Duncan’s test (*P <* 0.05).

Carbohydrate phloem loading primarily relies on two pathways, namely the symplastic and apoplastic pathways [24]. Compared with K0 treatment, K application increased the total cross-sectional area of vascular bundles at different stages; there were significant increases under K2 and K4 treatments at the stage of enlargement, under K4 treatment at the stage of color turning, and under K2 treatment at the mature stage (Fig. 5D–G, J and L). Further, we examined the symplastic pathway of carbon flow in phloem by using the symplastic tracer carboxyfluorescein diacetate (CFDA) staining method. When it is introduced into branches distant from the fruit, CFDA is cleaved into carboxyfluorescein (CF), which further diffuses through plasmodesmata [41]. Our results showed that CF signals were enhanced by K application, particularly at the stage of color turning (Fig. 5P and Q). Interestingly, the plasmodesmal density (PD) of SE (sieve element)–CC (companion cell), SE–PP (phloem parenchyma), and PP–PP cells in the leaves was increased by K application, and the PD of SE/CC and PP/PP cells was higher at the color turning stage than that at the enlargement and mature stages, which was consistent with the observation of CF signals in phloem in orange leaves (Table 1, Supplementary Data Figs S6–S8). The results indicated that K application improved carbon allocation from source leaves to fruit in Newhall orange, and that the color turning stage was a critical stage for phloem loading through the symplastic pathway.

**Table 1:** Effects of K fertilizer on plasmodesmal densities (number of plasmodesmata per μm cell length) between different cells in Newhall orange leaf vein.

Treatment	Enlargement stage				Turning stage				Mature stage			
	SE–CC	SE–PP	CC–PP	PP–PP	SE–CC	SE–PP	CC–PP	PP–PP	SE–CC	SE–PP	CC–PP	PP–PP
K0	0.39	0.46	0.30	0.20	0.87	0.33	0.49	0.31	0.46	0.04	0.10	0.24
K2	0.88	0.28	0.15	0.36	1.10	0.55	0.49	1.02	0.80	0.00	0.06	0.24
K4	1.05	0.48	0.13	0.24	0.67	0.32	0.20	1.32	0.55	0.25	0.41	0.32

The SUT is responsible for Suc transport in apoplastic loading and is also required for retrieving Suc in the apoplastic space [42, 43]. Our data showed that at the enlargement stage, the expression levels of *CsSUT1* and *CsSUT2* were significantly higher under K2 and/or K4 treatments than those under K0 treatment, and at the mature stage, the expression levels of *CsSUT1* were significantly higher under K2 treatment than those under K0 treatment (Fig. 6). This result suggested that K application promoted the expressions of *CsSUT1* and *CsSUT2* in citrus leaves.

## Discussion

### Appropriate potassium concentration improves fruit quality and accumulation of sugars in Newhall orange

K is an indispensable macronutrient in higher plants and it plays crucial roles in fruit quality of perennial fruit trees [4]. It was reported that K application increased soluble sugars accumulation in apple [44], pear [45], tomato [46], and grape [47]. Our data showed that K application increased the concentrations of soluble solids, Suc, Fru, and Glu in Newhall navel orange (Figs 1 and 2A–C), suggesting that K application might induce a series of changes related to sugar accumulation. In citrus fruit, sugar mainly consists of three major soluble sugars, namely, Suc, Glu, and Fru [48]. Moreover, the soluble sugar concentration in fruit is a crucial factor determining fruit quality [4, 49]. Therefore, the increased concentrations of soluble sugars induced by K application will contribute to improving fruit quality in Newhall orange. However, excessive K application decreased the sugar concentrations and fruit quality [50]. Generally, appropriate K supply is necessary to ensure plant growth and yield. For field crops, the K content in plant tissues ranges from 2 to 10% of dry matter [51]. However, the appropriate K contents in fruit are still to be quantified. In this study, our regression model showed that 1.5% of K in fruit was the optimal content (Supplementary Data Fig. S3); moreover, the K content above 1.5% would be detrimental to sugar accumulation and fruit quality. Taking these findings together, an appropriate K content improved fruit quality and sugar accumulation in Newhall navel orange fruit.

### Potassium application increases sugar accumulation by increasing sink and source strength in Newhall orange

Sink strength and source strength regulate carbon allocation, thus regulating the yield of crops and the fruit quality of horticultural plants [4, 52]. Sink strength can be quantified by the growth rate of organs [53]. Therefore, sink strength is affected by fruit size, number, and metabolic capacity, which affect carbon allocation in the fruits [54, 55]. In this study, we found that K application increased fruit size under K2 and K3 treatments and fruit number under K4 treatment (Supplementary Data Table S2). The average growth rate of sink organs under K treatments was higher than that without K application at enlargement, color turning and mature stages (Supplementary Data Table S2), which was consistent with one previous report that the increased growth rate of tomato fruit under far-red radiation contributed to the improvement of sink strength of individual fruits [55]. It has been reported that early flowering is a response to shading, possibly resulting in an elevated fruit number, thereby enhancing sink strength [56]. However, our data showed that appropriate K application induced only a slight increase in fruit number (Supplementary Data Table S2), indicating that K application increased sink strength not by increasing fruit number, which was in line with the finding that the increase in sink strength under far-red radiation did not depend on the increase in fruit number [55]. Therefore, the above results jointly suggest that K application enhances the sink strength of fruit by accelerating the growth rate, thereby facilitating the transport of carbohydrates into fruits, finally improving sugar accumulation and yield in fruits (Fig. 2A–C, Supplementary Data Table S2).

Sink strength is regulated by Suc metabolism. For example, it is regulated by multiple Suc-metabolizing enzymes, such as SPS, SS-S, SS-C, and INV [57]. This is supported by our results showing that the increased activities of SS-C, INV, and AI at the stages of enlargement and color turning and the increased expressions of *CsCINV* and *CsVINV* at the stage of enlargement in K treatments promoted the degradation of Suc from leaves, and thus generated a steep Suc gradient difference between source leaves and sink, indicating an enhanced sink strength (Fig. 2F–H, N, and O). Consistent with the findings of a previous study of tomato [50], our data showed that the enhanced activities of SPS and SS-S under K application resulted in Suc resynthesis from Suc-degraded products in citrus, and some Suc-degraded products have been reported to be utilized for energy metabolism, the carbon skeleton, and sugar signal [24]. Sink strength is also regulated by sugar transporter [52, 55]. In this study, we found that K application increased the expression of *CsSWEET15* (a gene responsible for sugar unloading in phloem) in Newhall orange fruit (Fig. 3), thus enhancing Suc efflux from phloem, which was supported by one previous study of tomato [58]. Subsequently, the degraded products of Suc, Fru and Glu were transported into vacuoles for storage by *CsSTP*s, *CsTMT*s, and *CsVGT*s [42]. Therefore, the increased expressions of *CsSTP11* and *CsTMT2* under K application at the stages of enlargement and color turning drove the sugars into vacuoles and hence increased the sink capacity of fruit to attract more carbohydrates into fruit at the early and mid-stages of fruit development. Taking these results together, K application improved sink strength by regulating sugar metabolism and transport, thus increasing sugar accumulation in Newhall orange navel fruit.

Source strength determines the amount of carbohydrate exported [39]. Unlike the sink strength of fruit, it is difficult to measure the source strength of leaves, but the capacity for carbon fixation and that for carbohydrate synthesis can reflect source strength [39, 52, 59]. Our results showed that K application enhanced net photosynthesis (*P*_n_) (Supplementary Data Table S3), indicating that K application improved carbon fixation, thus stimulating source strength. Additionally, Suc synthesis activity in leaves can reflect source strength [52]. In this study, K application increased the activities of Suc-synthesizing enzymes SPS and SS-S, but decreased AI activity in leaves at the color turning and mature stages (Supplementary Data Fig. S4), indicating that K treatments increased Suc synthesis and decreased Suc degradation, thus enabling source organs to supply more Suc to the sink organs, further creating a steeper Suc concentration gradient difference to drive carbon flow between source and sink in Newhall navel orange.

### Potassium stimulates carbon flow between source and sink and hence increases fruit sugar in Newhall orange

Previous studies have indicated that over half of the fruit sugars in citrus fruit originate from source leaves via phloem transport, suggesting that carbohydrate transport plays crucial roles in sugar accumulation in fruit [40, 57]. Our data demonstrated that K application enhanced source and sink strength (Figs 2 and 4), allowing source leaves to provide more carbohydrates and permitting the sink to provide a larger storage space, thus promoting the carbon flow between source and sink. Although K application enhanced carbon fixation and source activity, it failed to increase the concentrations of leaf soluble sugar (Fig. 4). With increasing soluble sugar concentrations, starch synthesis increases as the primary product in source leaves since K application can improve the activities of starch-synthesizing enzymes [60, 61]. However, our data showed that the concentrations of leaf starch were not elevated under K treatment (Fig. 4). The possible reason for such different findings might be that the increased carbohydrates were transported into fruit, which confirmed our observation that the concentrations of ^13^C-Suc and ^13^C-Glu in orange fruit were increased under K application (Fig. 5B and C).

Carbohydrates are initially loaded into the SE–CC complex in the phloem of leaves, transported over a long distance to the fruit, and finally unloaded on the segment epidermis of fruit by the symplastic or apoplastic pathway [62, 63]. Carbohydrate is transported into the CC predominantly via plasmodesmata in the symplastic pathway [64]. We found that the phloem loading of carbohydrates used the symplastic pathway in Newhall orange (Fig. 5N–S), as indicated by the symplastic tracer CFDA [41]. Moreover, we also found that the CF signal intensity was higher at the color turning stage than that at the enlargement and mature stages, and that under K application CF signal intensity was more significantly enhanced at the color turning stage compared with the enlargement and mature stages, indicating that the color turning stage played a more important role than the enlargement and mature stages in symplastic loading of carbohydrates, and that K application could enhance symplastic loading. Symplastic loading is a plasmodesmata-mediated process, and thus PD is of great significance for assessing the transport capacity of the symplastic pathway [31, 64, 65]. In accordance with the results for the symplastic tracer (Fig. 5N–S), the PD of SE–CC, CC–PP, and PP–PP cells was higher at the color turning stage than that at the enlargement and mature stages, and K application increased the PD of SE–CC cells at all stages under K2 treatment (Table 1, Supplementary Data Figs S6–S8), which further confirmed that phloem loading of carbohydrates was through the symplastic pathway at different stages, and this loading process was strengthened by K application.

The SUT is indispensable for the transport of Suc in the phloem, establishing the basis for the phloem loading of Suc and long-distance transport [31, 62]. Although we observed higher expressions of *CsSUT1* and *CsSUT2* in citrus leaves at the enlargement stage, this did not mean that Suc loading occurs through apoplastic loading, as SUTs also have the function of retrieving Suc from apoplast space [43, 66, 67]. We found obvious CF signal and numbers of plasmodesmata in the phloem of citrus leaves (Table 1, Fig. 5N–S, Supplementary Data Figs S6–S8). Moreover, the apoplastic pathway and the symplastic pathway are incompatible within the same sieve SE–CC complex [68], which implied that symplastic loading was more likely to be adopted than apoplastic loading in phloem loading of Suc in citrus. Consistent with our results, due to the persistently strong carbon sinks of tree plants and the relatively high photosynthetic efficiency, which implies that tree plants actually devoted a passive symplastic loading mechanism as it is less energy-consuming compared with active mechanism [68–70]. Taken together, our present results suggested that the phloem loading of Suc was carried out by symplastic loading in citrus.

After phloem loading, carbohydrates will be transported over a long distance to the fruit, and transport capacity is determined by carbon flow resistance, which mainly depends on the anatomical structure of phloem and the turgor pressure-mediated driving force [11, 13]. K application has been reported to increase the carbon transport rate in trunk phloem by increasing the cross-sectional area of sieve elements in eucalyptus trees [11]. Consistently, our results showed that K application increased the cross-sectional area of the vascular bundles of leaf veins. Moreover, K application increased K concentrations of phloem in Newhall orange branches (Supplementary Data Fig. S2), which was in line with a previous report on K concentrations in phloem sap of *Eucalyptus grandis* [71]. Importantly, the increased K concentrations in phloem were conducive to maintaining phloem pressure, thus generating a driving force for carbon flow [12]. Therefore, the cross-sectional area of vascular bundles and K concentrations contributed to driving carbon flow in K-treated plants, but the carbon transport rate in phloem remains to be further investigated in future work. Finally, carbohydrates will be unloaded on the segment epidermis of fruit mainly by the apoplastic pathway due to the higher expression of *CsSWEET15* (Fig. 3) and fewer plasmodesmata (unpublished data).

### Conclusions

**Figure 7:**
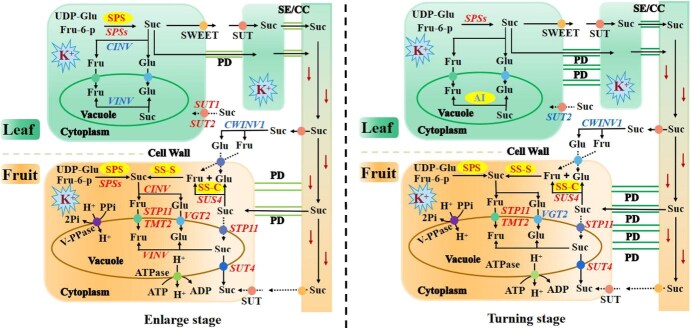
A comprehensive structure model of the effects of K on the expression of key genes and enzyme activities involved in Suc metabolism and transport in leaf and fruit in Newhall navel orange. Fru-6-p, fructose-6-phosphate; UDP-Glc, uridine diphosphate glucose; Fru, fructose; Glu, glucose; SPS, sucrose phosphate synthase; CWINV, cell wall invertase; CINV, cytoplasmic invertase; VINV, vacuolar invertase; SS-S, sucrose synthase synthesis direction; SS-C, sucrose synthase catalysis direction; SWEET, sugar will eventually be exported transporters; SUT, sucrose transporter; SE/CC, sieve element/ companion cell; VGT, tonoplast glucose transporter; STP, sugar transporter; TMT, tonoplast monosaccharide transporters; ATPase, adenosine triphosphatase; ATP, adenosine triphosphate; ADP, adenosine diphosphate; V-PPase, vacuolar H^+^-pyrophosphatase; Pi, phosphate; PPi, diphosphate. Red and blue fonts indicate upregulation and downregulation of enzyme activities or gene expression with increasing K application.

Taken together, our results indicate that the differences in sugar-metabolizing enzyme activities, vascular features, and sugar transporter functions led to a differential source–sink relationship at different K application levels in citrus (Fig. 7). K application enhanced photosynthesis, increased SPS activity and expressions of *CsSPS*s in source leaves, and raised SPS and SS activities and expressions of vacuole sugar transporter in fruit, thus inducing source–sink dynamics, eventually resulting in sugar accumulation in fruit. Additionally, the investigation results of the structural characteristics of leaf vein phloem cells, CF signal and a considerable number of plasmodesmata indicated that K application stimulated carbon flow through the symplastic pathway at different stages, ultimately promoting sugar accumulation. In summary, this study revealed the mechanism by which K application promoted sugar accumulation in citrus fruit by regulating the source–sink relationship, namely by enhancing source strength, sink activity, and carbon flow. Future research is suggested to quantify the contribution rates of the increased source strength, sink capacity, carbon flow, and metabolic flux of core metabolites under K application to sugar accumulation in citrus fruit.

## Materials and methods

### Experimental materials and design

A field experiment was conducted to study the effects of different amounts of K fertilizer application on soluble sugar accumulation and its mechanism by using 10-year-old Newhall navel orange (*C. sinensis*) grafted on trifoliate orange at the Citrus Research Institute of Ganzhou, Jiangxi province in 2020. The soil properties were as follows: pH, 4.35; organic matter, 14.43 g kg^−1^; alkaline hydrolysable N, 35.81 mg kg^−1^; Olsen-extractable P, 73.63 mg kg^−1^; exchangeable K, 183.76 mg kg^−1^. The experiment was set up with five K fertilizer treatments, K0, K1, K2, K3, and K4, in which the amount of K (K_2_O) applied was 0, 0.25, 0.5, 0.75, and 0.9 kg per plant, respectively; N fertilizer was applied at 0.8 kg per plant and P fertilizer (P_2_O_5_) at 0.4 kg per plant. These were provided with K_2_SO_4_, CO(NH_2_)_2_, and NH_4_H_2_PO_4_, respectively. Each treatment had four replicates with eight plants. The strategy of N, P, and K fertilizer application followed our previous studies with three split applications [4], namely bud fertilizer with 40% N, 60% P, and 30% K; fruit-preserving fertilizer with 30% N, 40% P, and 50% K; and fruit-expanding fertilizer with 30% N and 20% K. Twenty representative leaves and four fruits were randomly collected from the central periphery of the citrus crown at the stages of fruit enlargement (late July), fruit color turning (early October), and fruit mature (late November), and stored at −80°C for the analyses of carbohydrate*,* enzymes activities, and gene expression.

To confirm the effects of K fertilizer application on fruit soluble sugar accumulation, a pot experiment was carried out in Huazhong Agricultural University, Wuhan, Hubei province, using 3-year-old Newhall navel orange grafted on trifoliate orange. The soil properties were as follows: pH, 6.62; organic matter, 11.08 g kg^−1^; alkaline hydrolysable N, 65.19 mg kg^−1^; Olsen-extractable P, 18.82 mg kg^−1^; exchangeable K, 68.78 mg kg^−1^. Uniform orange seedlings were cultivated in plastic pots with 35 kg soil. During the period of the experiment, the pots were placed in a transparent rain-proof shed and watered as needed with distilled water. The experiment was set up with four K levels, K0, K1, K2, and K3, in which the application rates of K (K_2_O) were 0, 0.20, 0.40, and 0.60 g K_2_O kg^−1^ soil, and each treatment was replicated five times with five pots. The amounts of N and P fertilizer were CO(NH_2_)_2_ (as N) 0.2 g kg^−1^ soil and NH_4_H_2_PO_4_ (P_2_O_5_) 0.15 g kg^−1^ soil, respectively. The strategy of N, P, and K fertilizer was consistent with the field experiment. Fruits were collected at the mature stage for fruit quality analysis.

### Analysis of fruit quality

Fruits at the at each time period were selected to determined total soluble solid (TSS). The concentrations of TSS were determined by using a digital refractometer following a previous method [4].

### Analysis of soluble sugar and starch

Different stages of citrus leaves were ground with liquid nitrogen for the analysis of soluble sugar and starch. According to the method proposed by Ji *et al*. [55], homogenized samples of leaf were used to extract soluble sugar using 10 mL 80% (v/v) ethanol. Subsequently, the sample was centrifuged and the supernatant was used to measure soluble sugar. Next, 3 mL of distilled water was added to the precipitate, which was subsequently gelatinized in a boiling water bath for 15 min. Subsequently, 2 mL of precooled perchloric acid with a concentration of 9.2 mol L^−1^ was added, and after centrifugation the supernatant was utilized for starch determination.

### Photosynthetic data collection

Photosynthetic parameters [transpiration rate (*T*_r_); net photosynthetic rate (*P*_n_); intercellular CO_2_ (Ci); stomatal conductance (*G*_s_)] were measured using the Li-6400 Portable Photosynthesis Measurement System (LI-COR, Lincoln, Nebraska, USA), from 8:00 to 12:00 h. The photosynthesis instrument was set as follows: the CO_2_ concentration of the air in the chamber was controlled by the carbon dioxide cylinders, while light used for the measurements was supplied by the LI-6400 LED red/blue light source; the gas flow rate, the mixing fan speed, the humidity, and the air temperature of the leaf compartment were 10 000 revolutions min^−1^, 500 μmol s^−1^, 50%, and 25°C, respectively.

### Analysis of sugar concentrations

Frozen samples of fruit were ground with liquid nitrogen for sugar concentration determination, including sucrose (Suc), fructose (Fru), and glucose (Glu), according to our previous studies [50]. The homogenized samples were extracted with 80% methyl alcohol at 75°C for 15 min, then for ultrasonic extraction for 45 min, and centrifuged at 4000 × g for 10 min. After three extractions, 0.50 mL supernatant was dried by using a vacuum centrifugal concentration meter. After derivatization, 0.50 mL of supernatant was transferred into a 2-mL brown injection bottle and run on an Agilent 6890 N GC (Agilent, USA).

### ^13^C-Isotope labeling and analysis

According to Dominguez *et al*. [72], on the sunny side of the periphery of each tree crown, single fruit-bearing shoots with the same growth potential were selected for ring cutting and marked for feeding with ^13^CO_2_. Considering the effects of leaf-to-fruit ratio on leaf photosynthesis, all the fruit-bearing shoots were artificially adjusted to retain one marked mature leaf by picking leaves. The ^13^CO_2_ was supplied with NaH^13^CO_3_; one leaf was put into a plastic self-sealing bag containing 0.11 g NaH^13^CO_3_, and ^13^CO_2_ was released by injecting dilute H_2_SO_4_. After 6 h labeling, the fruit samples were harvested and stored at −80°C for the analysis of ^13^C-Suc, ^13^C-Fru, and ^13^C-Glu. Following derivatization, the supernatant was used to analyze ^13^C-Suc, ^13^C-Fru, and ^13^C-Glu by using a GC-IRMS (Agilent 7890B-Isoprime PrecisION). Isotope reference material was USGS67 (δ^13^ = −34.50‰), USGS67 (δ^13^ = −10.55‰), USGS67 (δ^13^ = −0.57‰) as standard, and ^13^C-Suc, ^13^C-Fru, and ^13^C-Glu were determined under the following conditions: DB-5MS column (50 m × 0.25 mm × 0.25 μm); inlet temperature 270°C; flow velocity 1.2 mL min^−1^; injection volume 1 μL.

### Microstructure observation and CFDA labeling

Plasmodesmata observations. The method of tissue preparation for ultrastructural observation was described by Chen *et al*. [73]. Single-fruit branches were selected at the enlargement, color turning, and mature stages of citrus fruits, and the leaf veins were cut transversely and incubated in 4% (v/v) glutaraldehyde for plasmodesmata observations under a transmission electron microscope (Hitachi, Japan). Plasmodesmata were counted at all cell interfaces, including the interfaces between sieve element (SE)–companion cell (CC), SE–phloem parenchyma cell (PP), CC–PP, and PP–PP in each selected field as described by Chen *et al*. [73].

Vascular bundle observations. The leaf veins mentioned above were incubated in FAA fixative for vascular bundle observations. The samples were cut into segments and then were used to make paraffin sections for observations of vascular bundle development with a microscope (Nikon, DS-Ri2) according to the methods of Chen *et al*. [73].

CFDA labeling. To examine Suc transport by the symplastic pathway in the phloem of citrus, a feeding experiment was conducted using 5(6)-carboxy fluorescein diacetate (CFDA) [73]. A singlefruit branch was labeled with 200 μL of 1 mg mL^−1^ CFDA aqueous solution, and select five branches from each tree. Citrus phloem was harvested after 48 h of *in vivo* transport of CFDA, and was selected for transverse and longitudinal cuts, sealed with 80% glycerol, and observed using a laser confocal microscope (Leica SP8, Germany) with an excitation wavelength of 488 nm.

### Analysis of enzyme activities related to sugar metabolism

Suc-metabolizing enzyme activities were measured according to the method of Wu *et al*. [4] with slight modification. Fruit samples of 1 g were homogenized in 8 mL precooled Suc-metabolizing enzyme extraction solution (pH = 7.5) containing 200 mM HEPES–NaOH buffer, 5 mM MgCl_2_, 0.1% β-mercaptoethanol, 0.05% Triton-X100, 0.05% BSA, 2% PVPP, 1 mM EDTA, 1 mM EGTA, 10 mM l-ascorbic acid sodium salt, 10 mM Cys–HCl, and 2% glycerin. Preparations were centrifuged at 10 000 revolutions min^−1^ for 30 min at 4°C, and the supernatant was separately supplemented with sucrose phosphate sucrose (SPS) reaction solution (50 mM HEPES–NaOH buffer, 5 mM NaF, 15 mM MgCl_2_, 1 mM EDTA, 0.1% β-mercaptoethanol, 15 mM G-6-P, 20 mM UDP-G, 4 mM F-6-P, pH = 7.5) and sucrose synthetase-synthesis (SS-S) reaction solution (80 mM HEPES–NaOH buffer, 5 mM NaF, 5 mM DTT, 15 mM UDP-G, 100 mM d-fructose, pH = 8.5), followed by determination of absorbance at 620 nm using anthrone colorimetry. Assays of the activity of sucrose synthetase cleavage (SS-C) used the 3,5-dinitrosalicylic acid reduction method. To the supernatant was added acid SS-C reaction solution (pH = 5.5) containing 80 mM MES buffer, 5 mM NaF, 5 mM UDP, and 100 mM sucrose. The preparation was incubated at a temperature of 30°C for 30 min, then DNS reagent was added, and the reaction was terminated by boiling in a water bath for 5 min. The OD value of each well plate was measured at a wavelength of 540 nm using an enzyme-linked immunosorbent assay instrument.

Acid invertase (AI) and neutral invertase (NI) activities were also measured according to the method of Wu *et al*. [4]. with slight modification. Fruit samples of 1 g were homogenized in 8 mL precooled invertase extraction solution (200 mM K_3_PO_4_ buffer, 5 mM MgCl_2_, 0.1% β-mercaptoethanol, 0.05% Triton-X100, 0.05% BSA, and 2% PVPP, pH = 7.5) on an ice bath for 10 min, followed by centrifugation at 10 000 revolutions min^−1^ for 30 min. The activities of AI and NI were determined following the SS-C assay, employing the 3,5-dinitrosalicylic acid reduction method; however, the AI reaction solution (pH = 5.5) contained 2 mM MgCl_2_, 1 mM EDTA, 100 mM sucrose, and 0.1 M CH_3_COOH-K_3_PO_4_ buffer, while the NI reaction solution (pH = 7.5) contained 2 mM MgCl_2_, 1 mM EDTA, 100 mM sucrose, and 0.1 M HEPES–NaOH buffer. The SS-C reaction solution (pH = 5.5) contained 80 mM MES buffer, 5 mM NaF, 5 mM UDP, and 100 mM Suc.

### RNA extraction and real-time PCR

Total RNA of leaf and fruit pulp were extracted using a Hipure Plant Total RNA kit (Magen Biotech, China) as described in the manufacturer’s protocol. After assessing RNA quality and quantity, the 1.0 μg of high-purity RNA was used to synthesize cDNA using a TRUEscript RT kit (Aidlab, China). Real-time PCR was performed using an ABI 7500 (Bio-Rad, USA) according to the instructions of MonAmp™ ChemoHS qPCR mix (Monad, China). Real-time PCR conditions were as follows: 95°C for 10 min followed by 40 cycles of 95°C for 10 s and 60°C for 30 s. *Actin* and *GAPDH* were used as reference genes, according to Zheng *et al*. [42] and Romero *et al*. [74], and the template primers used in this article are listed in Supplementary Data Table S1.

### Statistical analysis

Statistical analyses of data were performed using SPSS 20.0 applying Duncan’s multiple comparison test. The figures were drawn using Origin 2018 software.

## Supplementary Material

Web_Material_uhae240

## Data Availability

The data underlying this article are available in the article and in its online supplementary material.
