# Correction to: Potassium stimulates fruit sugar accumulation by increasing carbon flow in *Citrus sinensis*

**DOI:** 10.1093/hr/uhaf057

**Published:** 2025-03-04

This is a correction to: Kongjie Wu *et al.*, Potassium stimulates fruit sugar accumulation by increasing carbon flow in *Citrus sinensis, Horticulture Research*, Volume 11, Issue 11, November 2024, uhae240, 10.1093/hr/uhae240.

In the originally published version, *y*-axis units for soluble sugar concentration and starch concentration in Figure 4 were given as ‘$\% $’. The figure has been corrected to present these units as $ \textrm{mg}\cdot \text{g-1} $. This error does not impact the content, research results, or conclusions of the article.

