# Supplementary material for: Potassium stimulates fruit sugar accumulation by increasing carbon flow in Citrus sinensis
Source: Hortic Res. 2024 Sep 9;11(11):uhae240. doi: 10.1093/hr/uhae240 (PMC11540757; doi:10.1093/hr/uhae240)
Supplement: Web_Material_uhae240 [file web_material_uhae240.zip › Appendix.docx]

**Appendix**

**Figure S1** Effects of K application on K content of leaf, pericarp and pulp. (A) enlarge stage; (B) color turning stage; (C) mature stage. K0, K1, K2, K3 and K4 indicate that the citrus plants were cultivated in different K fertilizer levels of 0, 0.25, 0.5, 0.75, 0.9 kg K_2_O per plant under field culture. The data and error bars are the mean ± SE (n=4), and different lowercase letters represent significant differences among K treatments at the same stage by Duncan-test (*P <* 0.05).

**Figure S2** Effects of K application on K content of phloem. K0, K2 and K4 indicate that the citrus plants were cultivated in different K fertilizer levels of 0, 0.5, 0.9 kg K_2_O per plant under field culture. The data and error bars are the mean ± SE (n=4), and different lowercase letters represent significant differences among K treatments at the same stage by Duncan-test (*P <* 0.05).

**Figure S3** The fitting curve of K application levels and K concentrations and sugar composition at mature stage.

**Figure S4** Effects of K fertilizer on sucrose metabolism in Newhall navel orange leaf. (A) sucrose phosphate synthase (SPS); (B) Sucrose synthetase-synthesis (SS-S); (C) Sucrose synthetase-cleavage (SS-C); (D) Acid invertase (AI); (E) Neutral invertase (NI). K0, K2 and K4 indicate that the citrus plants were cultivated in different K fertilizer levels of 0, 0.5, 0.9 kg K_2_O per plant under field culture. The data and error bars are the mean ± SE (n=4), and different lowercase letters represent significant differences among K treatments at the same stage by Duncan-test (*P <* 0.05).

**Figure S5** Effects of K fertilizer on the expressions of sugar transporters in Newhall navel orange leaf. K0, K2 and K4 indicate that the citrus plants were cultivated in different K fertilizer levels of 0, 0.5, 0.9 kg K_2_O per plant under field culture. The data and error bars are the mean ± SE (n=4), and different lowercase letters represent significant differences among K treatments at the same stage by Duncan-test (*P <* 0.05).

**Figure S6** Ultrastructure of the sieve element-companion cell complex and its surrounding phloem parenchyma cells of Newhall orange leaf vein at the enlargement stage. SE, sieve element; CC, companion cell; PP, phloem parenchyma cells; PD, Plasmodesmata. K0, K2 and K4 indicate that the citrus plants were cultivated in different K fertilizer levels of 0, 0.5, 0.9 kg K_2_O per plant under field culture.


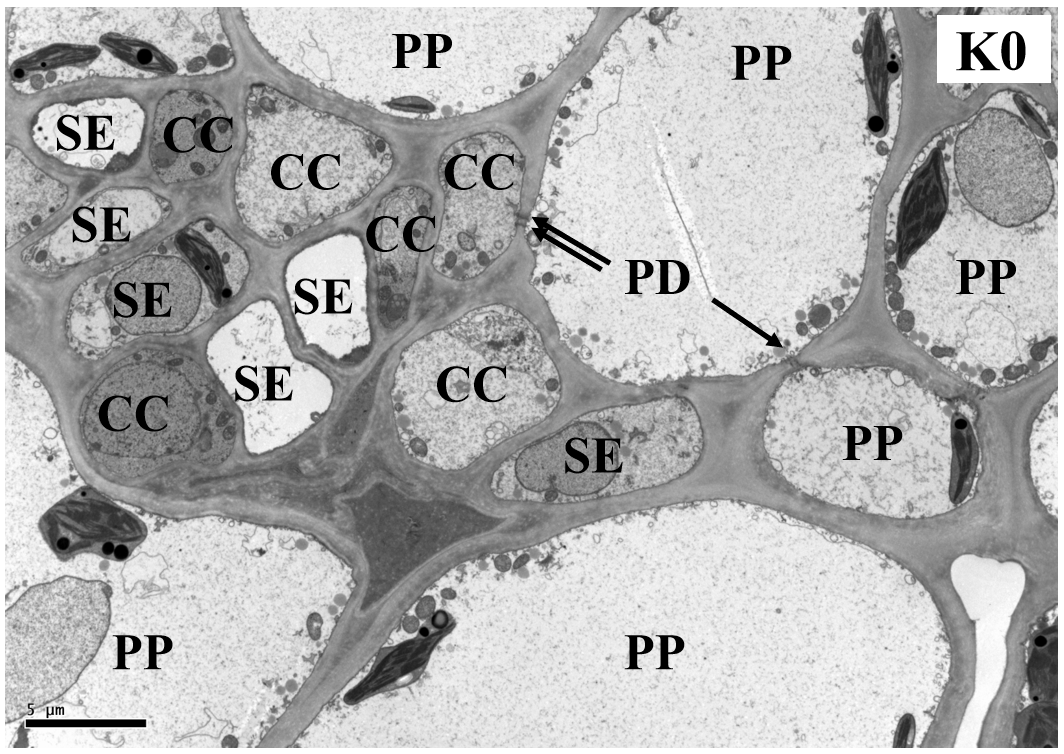


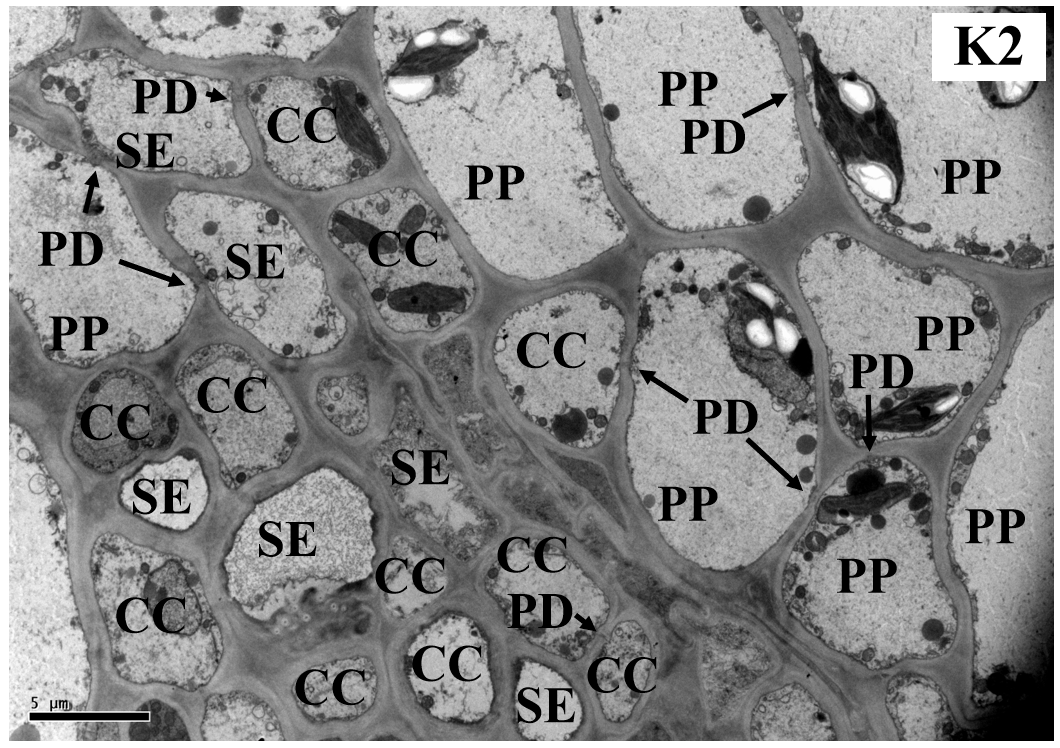


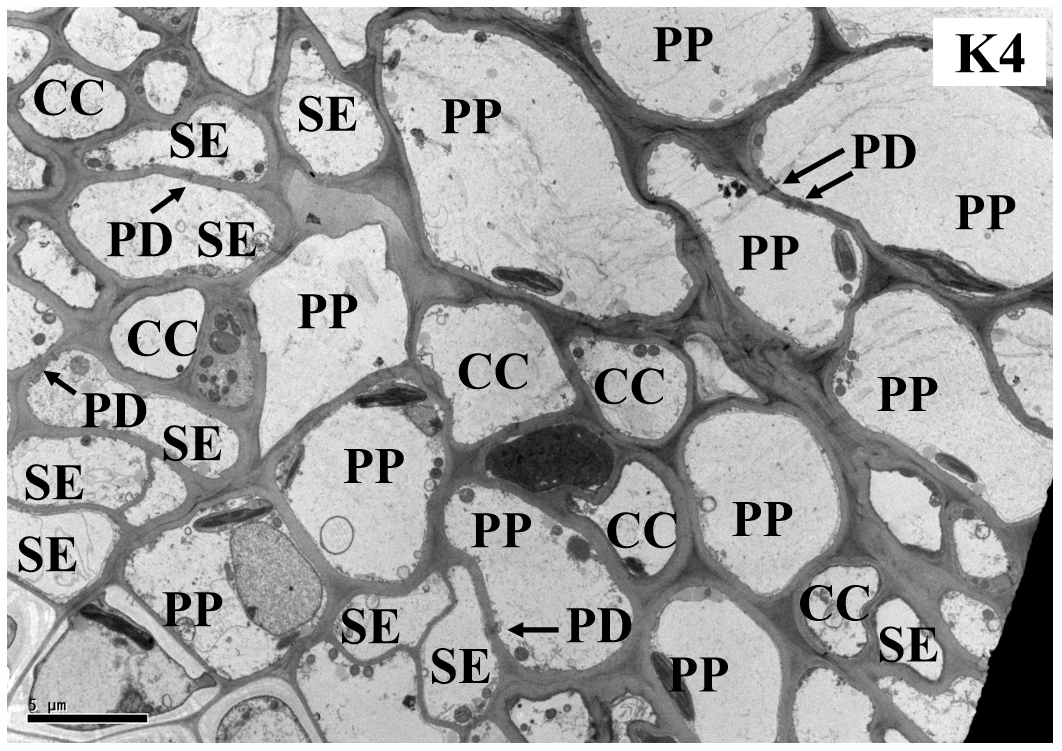


**Figure S7** Ultrastructure of the sieve element-companion cell complex and its surrounding phloem parenchyma cells of Newhall orange leaf vein at the color turning stage. SE, sieve element; CC, companion cell; PP, phloem parenchyma cells; PD, Plasmodesmata. K0, K2 and K4 indicate that the citrus plants were cultivated in different K fertilizer levels of 0, 0.5, 0.9 kg K_2_O per plant under field culture.


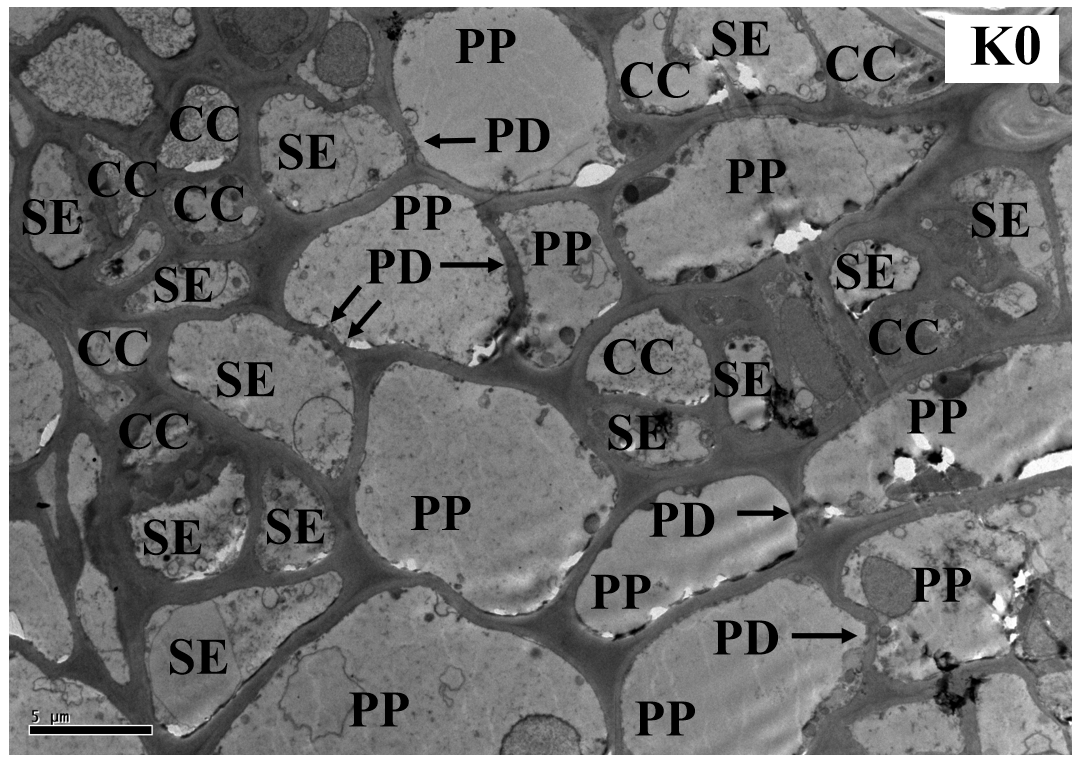


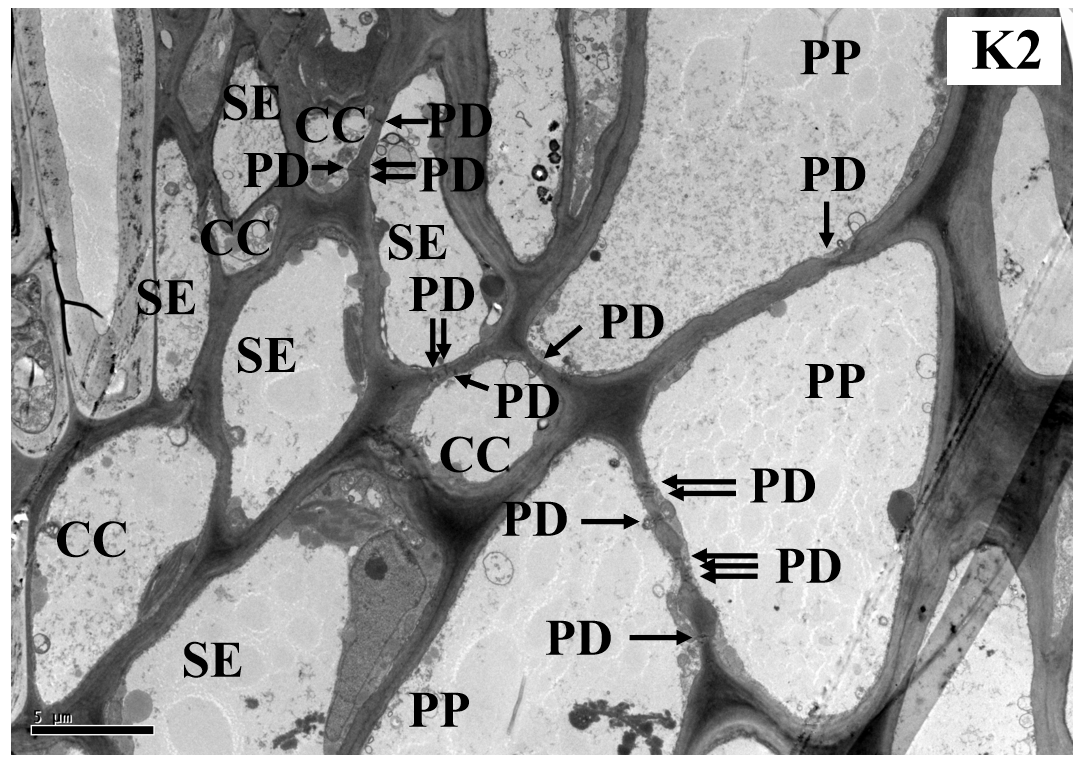

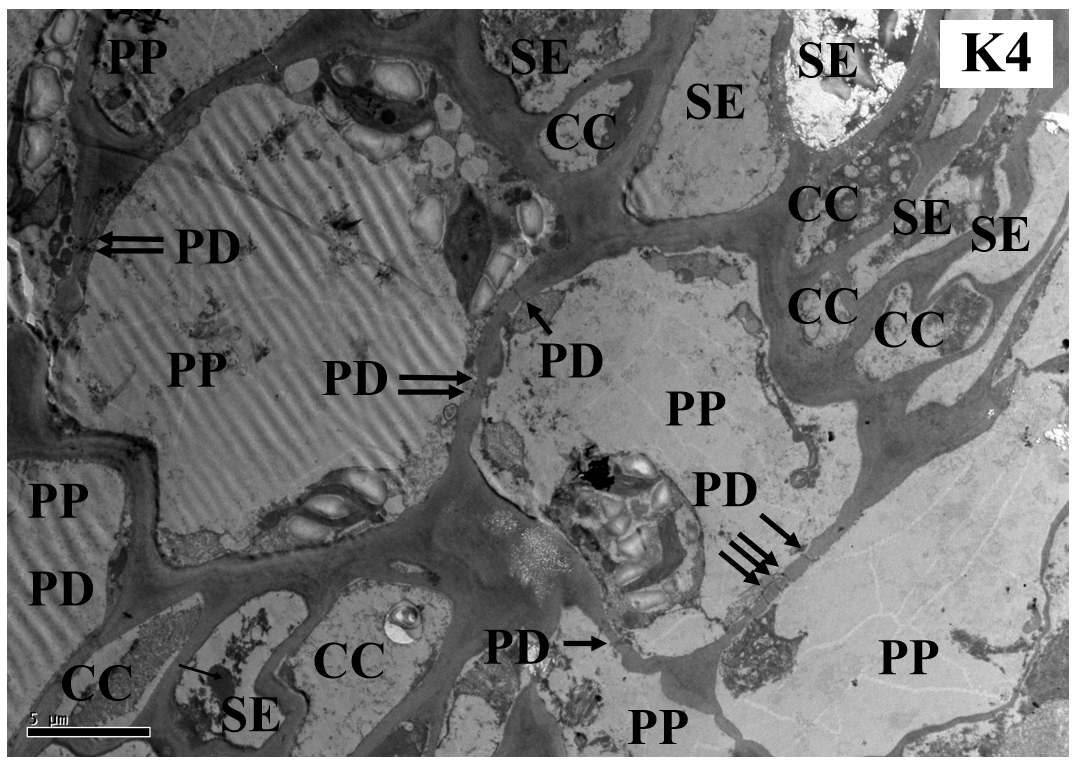


**Figure S8** Ultrastructure of the sieve element-companion cell complex and its surrounding phloem parenchyma cells of Newhall orange leaf vein at the mature stage. SE, sieve element; CC, companion cell; PP, phloem parenchyma cells; PD, Plasmodesmata. K0, K2 and K4 indicate that the citrus plants were cultivated in different K fertilizer levels of 0, 0.5, 0.9 kg K_2_O per plant under field culture.


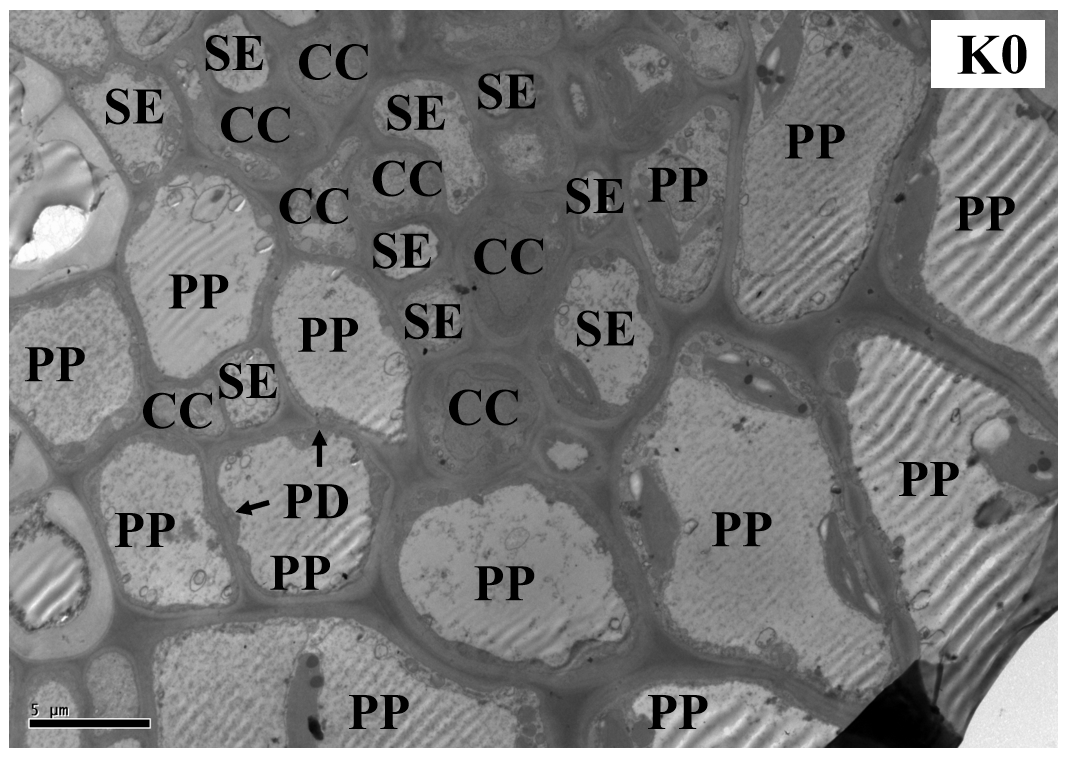


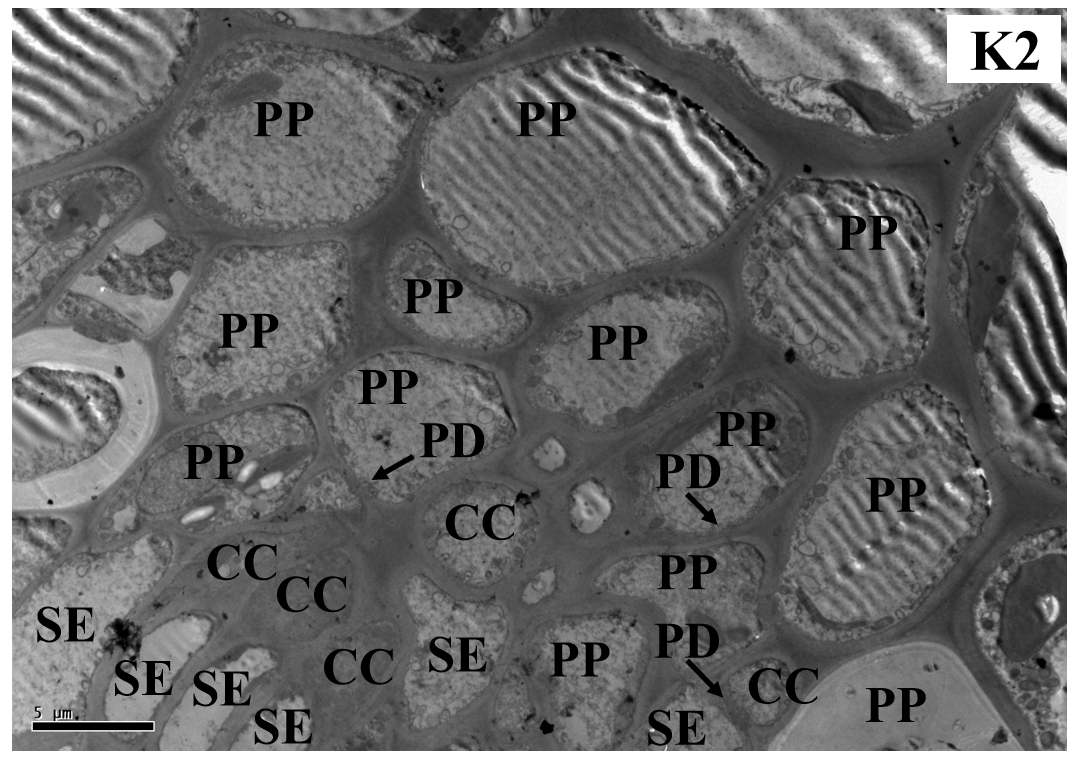

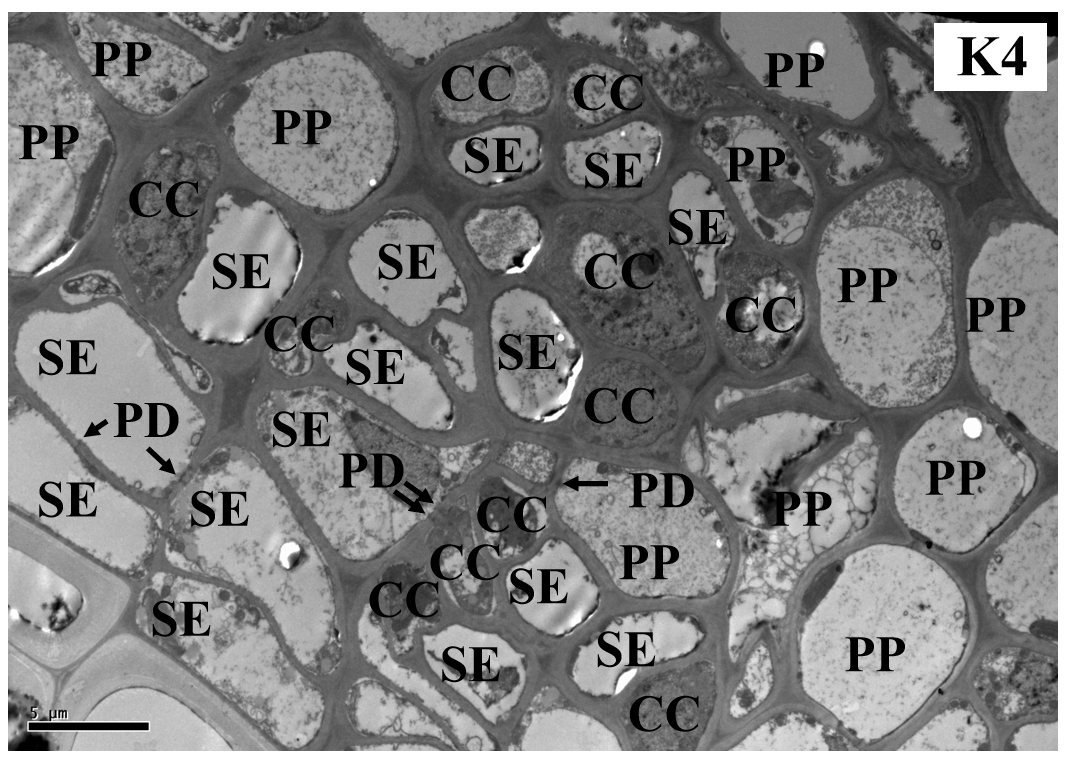


**Table S1** Primers used in this study

| Annotation | Gene name | Prime | | Reference |
| --- | --- | --- | --- | --- |
|  |  | Forward primer | Reverse primer |  |
| Vacuolar invertase | *VINV* | GGTGTTACATGGCGTCCCTA | TGGCACCCATGTCACATTCT | ^67^ |
| Cytosolic Invertase | *CINV* | TGGGAGAAGACTGTGGACTG | GCAACACGACCAATGGCTGA | ^10^ |
| Cell wall invertase | *CWINV1* | CAAAAGGAGCAGTATGGGGC | AGGATTGTGGCTGAACCCGA | ^10^ |
| Sucrose Synthase | *SUS4* | GCTGGAAGTAAGGATAGGCC | CAGATTGATCAGCCCCAGGG | ^10^ |
| Sucrose Phosphate Synthase | *SPS1* | AAATTGGTGGTGGGAAGCCA | CGGATAGAAGGGCAGCTGAG | ^10^ |
|  | *SPS2* | TATTTGTGCAGTCTTCATGGCTT | AACATACTTAACCTGACCCCCTGT | ^10^ |
|  | *SPS3* | GGCCGGAAACGAGTGGATAA | TGCCAAGTTCACTGGGGTTT | ^10^ |
|  | *SPS4* | CTTGAATGTGCCGATGGTGC | GAAGCCTGCCTTGTTTCAGC | ^68^ |
| Tonoplast Monosaccharide Transporter | *TMT1* | CCACCAAGGCTGACTAATCGA | AGCTAATTCTGCCGGCTAAGC | ^38^ |
|  | *TMT2* | TCATCAAAGTTCCAGAAACAAAGG | CAACGGCGAAGAACTCTGTAATC | ^38^ |
| Vacuolar glucose transporter | *VGT1* | GTGGAATTTCTTGGTATGGCTTAT | CCCAGAATGTCGGCAATGTTA | ^38^ |
|  | *VGT2* | TTTCTTATCCACCAAGCCATTACT | ATGTCATACCCAAACAACAATCCT | ^38^ |
| Sugar transporter | *STP7* | GGGATGGAGGCTCTCACTTG | TTCCTCCCACTGTCATCATTAAAG | ^38^ |
|  | *STP11* | CAGCCTAATTCAGCGCAAGTC | CACGCGTTGCAGCATCAG | ^38^ |
| Sucrose transporter | *SUT1* | TGCAACCGCAAATCAAACTAAT | TGTCCCTTCTTTTCTTCCAACAA | ^38^ |
|  | *SUT2* | CCCATCAGCCATCCCTTAAG | GATTTCCGCAGGGTGTTAAGC | ^38^ |
|  | *SUT4* | ACGTGCAAGAGCTCGGAATT | GGCCACAGAGCCAGATGATAC | ^38^ |
|  | *SWEET15* | TATCTCGTTCCTCGTGTACCT | AGCCAGCGTTACTGGATTATT | ^38^ |
|  | *SWEET16* | GCCGCTTTGCAGTTCATTTA | CCGAGGAAGCCAACATCTAATA | ^38^ |
| Actin | *Actin* | CCAAGCAGCATGAAGATCAA | ATCTGCTGGAAGGTGCTGAG | ^38^ |
| GAPDH | *GAPDH* | CGTCCCTCTGCAAGATGACTCT | GGAAGGTCAAGATCGGAATCAA | ^66^ |

**Table S2** Effects of K fertilizer under filed culture

| Treatment | Single fruit weigh（g） | Fruit number per plant | Fruit growth rate | Average yield per plant（kg） |
| --- | --- | --- | --- | --- |
| K0 | 261.24±7.34b | 85.2±12.34b | 1.09±0.03b | 23.38±3.99b |
| K1 | 264.71±5.97ab | 95.71±13.31b | 1.10±0.02ab | 25.39±3.43b |
| K2 | 273.29±7.38a | 96.88±10.53b | 1.14±0.03a | 26.55±2.89b |
| K3 | 274.25±5.93a | 98.43±10.52b | 1.14±0.24a | 27.10±2.90b |
| K4 | 265.01±8.17ab | 117.33±17.42a | 1.10±0.03ab | 32.08±4.76a |

Note: The data and error bars are the mean ± SD (n=4), and different lowercase letters represent significant differences among K treatments at same stage by Duncan-test (*P <* 0.05).

**Table S3** Effect of K fertilizer on photosynthesis in citrus leaf

| Treatment | Tr (mmol· m^-2^·s^-1^) | Pn (µmol· m^-2^·s^-1^) | Ci (µmol mol^-1^) | Gs (mol· m^-2^·s^-1^) |
| --- | --- | --- | --- | --- |
| K0 | 0.75±0.29b | 5.12±1.36c | 228.99±25.53a | 0.05±0.02c |
| K1 | 1.30±0.39a | 7.49±1.30b | 240.21±47.39a | 0.09±0.03ab |
| K2 | 1.34±0.41a | 8.36±2.04ab | 238.08±38.05a | 0.10±0.03a |
| K3 | 0.99±0.27b | 7.33±1.39b | 213.47±18.07b | 0.07±0.02bc |
| K4 | 0.95±0.40b | 9.45±2.08a | 181.92±43.27b | 0.07±0.03bc |

Note: Tr, transpiration rate; Pn, net photosynthetic rate; Ci, intercellular CO_2_; Gs, stomatal conductance. The data and error bars are the mean ± SD (n=4), and different lowercase letters represent significant differences among K treatments at same stage by Duncan-test (*P <* 0.05).
